# Disease-causing mutations in genes encoding transcription factors critical for photoreceptor development

**DOI:** 10.3389/fnmol.2023.1134839

**Published:** 2023-04-27

**Authors:** Chi Sun, Shiming Chen

**Affiliations:** ^1^Department of Ophthalmology and Visual Sciences, Washington University in St. Louis, St. Louis, MO, United States; ^2^Department of Developmental Biology, Washington University in St. Louis, St. Louis, MO, United States

**Keywords:** photoreceptor development, transcription factor, mutation, inherited retinal disease, pathogenic mechanism

## Abstract

Photoreceptor development of the vertebrate visual system is controlled by a complex transcription regulatory network. OTX2 is expressed in the mitotic retinal progenitor cells (RPCs) and controls photoreceptor genesis. CRX that is activated by OTX2 is expressed in photoreceptor precursors after cell cycle exit. NEUROD1 is also present in photoreceptor precursors that are ready to specify into rod and cone photoreceptor subtypes. NRL is required for the rod fate and regulates downstream rod-specific genes including the orphan nuclear receptor NR2E3 which further activates rod-specific genes and simultaneously represses cone-specific genes. Cone subtype specification is also regulated by the interplay of several transcription factors such as THRB and RXRG. Mutations in these key transcription factors are responsible for ocular defects at birth such as microphthalmia and inherited photoreceptor diseases such as Leber congenital amaurosis (LCA), retinitis pigmentosa (RP) and allied dystrophies. In particular, many mutations are inherited in an autosomal dominant fashion, including the majority of missense mutations in *CRX* and *NRL*. In this review, we describe the spectrum of photoreceptor defects that are associated with mutations in the above-mentioned transcription factors, and summarize the current knowledge of molecular mechanisms underlying the pathogenic mutations. At last, we deliberate the outstanding gaps in our understanding of the genotype–phenotype correlations and outline avenues for future research of the treatment strategies.

## Introduction

Transcription factors regulate the cell-type specification and differentiation in the retina (Livesey and Cepko, 2001; Harada et al., 2007; Hennig et al., 2008; Byerly and Blackshaw, 2009; Swaroop et al., 2010; Heavner and Pevny, 2012; Brzezinski and Reh, 2015; Stenkamp, 2015; Miesfeld and Brown, 2019; Seritrakul and Gross, 2019; Raeisossadati et al., 2021). Retinal development is highly conserved among vertebrates (Stenkamp, 2015). This review firstly summarizes the up-to-date knowledge of functions of selected transcription factors involved in early stages of photoreceptor development. These transcription factors include OTX2, CRX, NEUROD1, NRL, NR2E3, THRB, and RXRG (Table 1). Secondly, this review describes the congenital disorders that result when these transcription factors are disrupted. Lastly, this review introduces ocular diseases that are associated with distinct forms of mutations in transcription factor genes such as *PRDM13* and *RAX2*.

**Table 1:** Selected transcription factors in this review.

Transcription factor	Primary function	Model organism	Note	Comment	Notable interaction	Remarkable ocular disease
OTX2	Optic vesicle formation (Adler and Canto-Soler, 2007)	Mouse			CRX, Vsx2 (Chx10), Prdm1 (BLIMP1), TLE4 (Samuel et al., 2014; Chan et al., 2020; Torero Ibad et al., 2020; Yamamoto et al., 2020)	Anophthalmia, microphthalmia
	RPE specification (Martinez-Morales et al., 2001)	Mouse				
	RPC specification (Trimarchi et al., 2008; Emerson and Cepko, 2011; Muranishi et al., 2011; Buenaventura et al., 2018)	Mouse				
	Photoreceptor genesis (Nishida et al., 2003)	Mouse	*pCrx-Cre*	*Otx2* deficiency re-specifies photoreceptor precursors into amacrine precursors (Nishida et al., 2003; Sato et al., 2007; Yamamoto et al., 2020).		
	Bipolar cell genesis (Koike et al., 2007)	Mouse	*pPcp2/L7-Cre*	*Otx2* overexpression results in ectopic genesis of photoreceptors at the expense of bipolar cells (Nishida et al., 2003; Yamamoto et al., 2020).		
	Horizontal cell genesis (Sato et al., 2007)	Mouse	*pDkk3-Cre*			
CRX	Photoreceptor development (Furukawa et al., 1999; Bibb et al., 2001; Chen et al., 2002; Plouhinec et al., 2003; Shen and Raymond, 2004; Nelson et al., 2008; Glubrecht et al., 2009; Ruzycki et al., 2018)	Mouse, Zebrafish, Cat, Amphibian	*Crx−/−* (Mouse)	Photoreceptor differentiation is disrupted in *Crx−/−* retina (Tran and Chen, 2014).	CBP, P300, NRL, NR2E3 (Peng et al., 2005; Peng and Chen, 2007; Corbo et al., 2010; Hennig et al., 2013)	LCA, RP, CRD
NEUROD1	Photoreceptor development (Yan and Wang, 1998; Morrow et al., 1999; Pennesi et al., 2003; Akagi et al., 2004; Yan and Wang, 2004; Wang and Harris, 2005; Cho et al., 2007; Ochocinska et al., 2012)	Mouse, zebrafish, chicken, amphibian	*pCrx-Cre* (Mouse)	*NeuroD1* embryonic knockout in C57BL/6 J mice causes lethal neonatal diabetes (Naya et al., 1997).	TRb2 (Liu et al., 2008)	RP
NRL	Rod photoreceptor development (Mears et al., 2001; Daniele et al., 2005; Nikonov et al., 2005; McIlvain and Knox, 2007; Montana et al., 2011; Kim et al., 2016; Oel et al., 2020; Cuevas et al., 2021)	Mouse, zebrafish, amphibian	*Nrl−/*− (Mouse)	*Nrl−/−* retina lacks rod photoreceptors but develops cone-like photoreceptors (Daniele et al., 2005; Nikonov et al., 2005).	CRX, NR2E3 (Hao et al., 2012; Liang et al., 2022)	ESCS, RP
NR2E3	Rod photoreceptor development (Haider et al., 2000; Milam et al., 2002; Cheng et al., 2004; O’Brien et al., 2004; Chen et al., 2005; Cheng et al., 2006; Haider et al., 2006; Cheng et al., 2011; Xie et al., 2019)	Mouse, zebrafish	*rd7* (Mouse)	The number of *Opn1sw*-expressing photoreceptors doubles in *rd7* mice (Corbo and Cepko, 2005).	CRX, NRL	ESCS, RP
THRB	Cone photoreceptor development (Ng et al., 2001; Suzuki et al., 2013; Eldred et al., 2018; Aramaki et al., 2022)	Mouse, zebrafish	*Thrb−/−* (Mouse), *pTrβ2-Cre* (*Mouse*), *thrb−/−* (zebrafish)	*Thrb2^−/−^* mouse retina shows decreased *Opn1mw* expression and increased *Opn1sw* expression (Ng et al., 2001).		Retinal defects associated with RTHβ.
RXRG	Cone photoreceptor development (Hoover et al., 1998; Janssen et al., 1999; Mori et al., 2001; Cossette and Drysdale, 2004; Roberts et al., 2005; Stevens et al., 2011).	Mouse, zebrafish, chicken, amphibian	*Rxrg−/−* (Mouse)	*Rxrg−/−* mouse retina shows increased *Opn1sw* expression (Roberts et al., 2005). *Rxrga* expression is also found in zebrafish rod photoreceptors (Sun et al., 2018).	RAR (Cvekl and Wang, 2009; Dawson and Xia, 2012)	

CRD, cone-rod dystrophy; ESCS, enhanced s-cone syndrome; LCA, Leber congenital amaurosis; RP, retinitis pigmentosa; RPC, retinal progenitor cell; RPE, retinal pigment epithelium; RTHβ, resistance to thyroid hormone beta.

*OTX2* expression is enriched in a large population of retinal progenitor cells, which determines photoreceptor genesis (Ghinia Tegla et al., 2020). *CRX* and *NEUROD1* are expressed in the photoreceptor precursors (Morrow et al., 1999; Hennig et al., 2008; Swaroop et al., 2010). Subsequently, these precursors are fated into rod and cone photoreceptors. Rod lineage is governed by rod-specific transcription factors such as NRL and NR2E3 (Mears et al., 2001; Milam et al., 2002); cone lineage is regulated by transcription factors such as THRB and RXRG (Ng et al., 2001; Deeb, 2006). A precise regulation on the expression of these transcription factors is essential for neurogenesis, cell survival, and homeostasis of photoreceptors. Targetome analysis also helps to determine the overall transcription factor networking involved in photoreceptor development. Therefore, the aberrant or ablated expression of each transcription factor or the networking always results in photoreceptor underdevelopment and degeneration. Mutations in the coding regions of these transcription factors may induce misregulation in target gene expression, thus produce blindness-causing retinopathies, including microphthalmia, Leber congenital amaurosis, retinitis pigmentosa, and cone-rod dystrophy. This review attempts to unveil the relationship between mutations, protein functions and disease phenotypes, and classify (or ‘re-classify) noteworthy mutations of each transcription factor based on mutant protein functions and resulted ocular phenotypes. Interestingly, many cases of missense mutations within the DNA-binding domains, including some *CRX* and *NRL* mutations show reduced DNA-binding capabilities and altered binding motif preference or affinity at specific sites. On the other hand, mutations within the coding regions of activation domains or domains that carry regulatory activity often downregulate the expression of target genes, with some exceptional cases. Moreover, a significant number of disease-causing mutations, regardless of the locations in the coding regions, belong to the autosomal dominant class. This review describes several examples to illustrate the potential pathogenic mechanisms.

### Selected transcription factors involved in early stage of photoreceptor development and diseases

#### OTX2

Photoreceptor development starts from the fate specification of the progenitor pool. *OTX2*, a homeobox gene located on human chromosome 14, encodes a key transcription factor for the development of nervous systems, including brain and retina specification (Acampora et al., 1995; Matsuo et al., 1995; Ang et al., 1996; Cantos et al., 2000; Henderson et al., 2009; Béby et al., 2010; Bernard et al., 2014). OTX2 function in retinal development is briefly introduced in Table 1. *OTX2* may function as an oncogene during development. *OTX2* overexpression is detected in retinoblastoma (Glubrecht et al., 2009; Li et al., 2015). Pharmacologic inhibition by all-trans retinoic acid (ATRA) reduces *OTX2* expression, therefore decreases cell proliferation and tumor growth (Li et al., 2015). *OTX2* overexpression is also found in some cases of medulloblastoma, repressing transcription of differentiation markers (Bunt et al., 2012; Lu et al., 2017). OTX2 can directly activate c-*MYC* expression in medulloblastoma *via cis*-regulatory elements in *MYC* promoter (Adamson et al., 2010; Bunt et al., 2011). Notably, concurrent trilateral retinoblastoma and medulloblastoma has been reported (Elias et al., 2001; Jurkiewicz et al., 2010), and aberrant *OTX2* expression is a common characteristic.

In a mature retina, cell identity no longer requires *Otx2* expression. *Otx2* is weakly expressed in rod and cone photoreceptors (Koike et al., 2007), strongly in bipolar cells (Fossat et al., 2007; Kim et al., 2008; Aavani et al., 2017), and in some Müller glia (Brzezinski et al., 2010), regulating their functions by cell-autonomous or non-autonomous actions (Housset et al., 2013; Torero Ibad et al., 2020). *Otx2* expression is required for the long-term survival of rod and cone photoreceptors, bipolar cells, and horizontal cells (Béby et al., 2010; Housset et al., 2013). Photoreceptor-specific *Otx2* conditional knockout after photoreceptor differentiation induces impaired translocation of arrestin-1 as well as downregulation of ECM components including versican and decorin in the retina (Pensieri et al., 2021). Similarly, in the visual cortex, OTX2 binds to regulate chondroitin sulfate proteoglycans of perineuronal nets (Beurdeley et al., 2012; Bernard et al., 2016), supporting the association of OTX2 with ECMs and cytoskeletons (Boncinelli and Morgan, 2001).

Photoreceptor-specific *Otx2* conditional knockout after photoreceptor differentiation does not alter the short-term retinal structure and phototransduction activity (Pensieri et al., 2021), which is thought to be compensated by *Crx* expression. Another piece of evidence is that loss of OTX2 in *Crx−/−* photoreceptors worsens the degenerative phenotypes (Hsiau et al., 2007). A possible explanation is that the optimal OTX2-binding site contains the 5′-TAAT-3′ sequence which is recognized by many other homeobox transcription factors such as CRX (Chen et al., 1997; Chatelain et al., 2006; Samuel et al., 2014). Tissue-specificity of transcription regulation is determined by unique sequences flanking this tetranucleotide (Berger et al., 2008; Jolma et al., 2015), not by the bound transcription factors. Such compensatory regulation between OTX2 and CRX is subjected to further investigation.

Interestingly, OTX2 can be transferred to cells that do not express it (Lee et al., 2019; Di Nardo et al., 2020). Exogenous OTX2 promotes the neuroplasticity of the visual cortex (Sugiyama et al., 2008) and survival of retinal ganglion cells and bipolar cells (Torero Ibad et al., 2011; Kim et al., 2015) by transcription regulation (Apulei et al., 2019) or mitochondrial energy complex stabilization (Kim et al., 2015). A proteomic analysis confirms the association of OTX2 with proteins of the mitochondrial energy complex as well as with the neurotransmitter machinery in the retina (Fant et al., 2015). Notably, this type of OTX2 transfer appears to be directional: OTX2 found in type2-off bipolar cells is transferred from photoreceptors (Kim et al., 2015); OTX2 found in ganglion cells is probably transferred from bipolar cells or photoreceptors (Sugiyama et al., 2008); OTX2 found in outer segments of photoreceptors is transferred from RPEs (Pensieri et al., 2021). This phenomenon reflects that OTX2 transfer between retinal cells probably contributes to the non-autonomous action of OTX2 regulating the retinal physiology.

**Figure 1:**
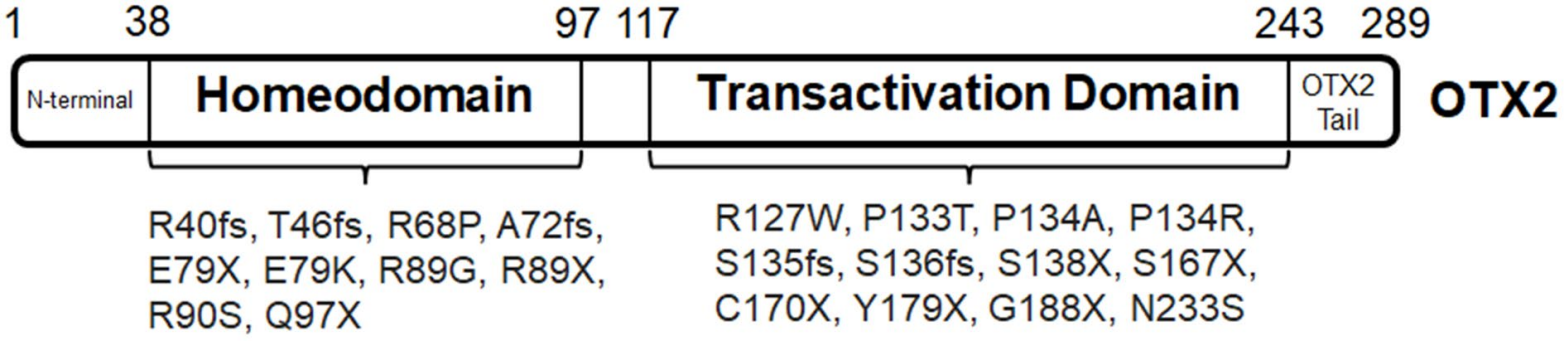
OTX2 protein domains and associated mutations.

The OTX2 protein has four major domains, namely, a N-terminal domain, a homeodomain, a C-terminal domain, and conserved OTX tail (Figure 1). The homeodomain (location: aa38–97) is a conserved 60-amino acid domain that binds to specific genomic targets (Di Nardo et al., 2018). C-terminal domain is also known as transactivation domain, consisting of nuclear localization signal and transcription regulatory region. In general as shown in reporter assays, OTX2 proteins lacking homeodomain are inactive in DNA-binding, and those lacking the C-terminal domain lose most of the transactivation capacity (Chatelain et al., 2006). In addition, the post-translational modifications of the OTX2 protein are largely unclear. A piece of corroborative evidence presents that interaction between OTX2 and TLE1 is governed by OTX2 phosphorylation during eye formation in xenopus (Satou et al., 2018).

Haploinsufficiency for OTX2 with only a single copy of a coding allele causes microphthalmia in mouse models (Matsuo et al., 1995; Kim et al., 2015) and rare human cases (Wyatt et al., 2008; Tajima et al., 2009). Heterozygous *OTX2* mutations in human patients result in severe ocular malformations which are usually associated with abnormal development in brain and pituitary dysfunction (Beby and Lamonerie, 2013). The clinical manifestations include unilateral and bilateral forms of anophthalmia/microphthalmia, optic nerve hypoplasia and coloboma (Ragge et al., 2005; Gorbenko Del Blanco et al., 2012). Notably, *OTX2* mutations are linked to the etiology of 2–3% of anophthalmia/microphthalmia cases (Wyatt et al., 2008; Tajima et al., 2009; Jones et al., 2016).

There is no clear genotype–phenotype correlation for *OTX2* mutations. Associations can however be proposed between disease phenotypes and domains of the mutant proteins. Firstly, it is worth noting that a large majority of mutations within the coding region for homeodomain including *OTX2^R40GfsX47^*, *OTX2^T46NfsX42^*, *OTX2^R68P^*, *OTX2^A72HfsX15^*, *OTX2^E79X^*, *OTX2^R89G^*, *OTX2^R89X^*, *OTX2^R90S^,* and *OTX2^Q97X^* causes bilateral microphthalmia (Ragge et al., 2005; Ashkenazi-Hoffnung et al., 2010; Gonzalez-Rodriguez et al., 2010; Schilter et al., 2011; Gregory et al., 2021). The functional assays show absent or nearly lost transactivation activity. These mutations generally cause frameshifts or premature stop codons producing mutant proteins with largely truncated or missing transactivation domain. In addition, many of these mutations carry no dominant-negative effect based on the functional analysis of the mutation proteins in cultured cells. Thus, the neuronal disorders are predicted as a result of OTX2 haploinsufficiency. Only few missense mutations have been reported so far. Seven patients of two families carrying the same missense mutation *OTX2^E79K^* show pattern dystrophy of RPEs at macula with normal or moderately reduced rod-driven or cone-driven electroretinogram (ERG) responses (Vincent et al., 2014). It is unclear if the dominant *OTX2^E79K^* (c.235G > A) carries any DNA-binding specificity and transactivation capacity in the retina and why the manifestations in *OTX2^E79K^* patients (Vincent et al., 2014) are different from those with *OTX2^E79X^* (c.235G > T) (Gregory et al., 2021).

Secondly, mutations within the coding region for transactivation domain produce variable disease phenotypes. Notably, *OTX2^P133T^* (bilateral microphthalmia), *OTX2^P134A^* (unilateral anophthalmia), *OTX2^P134R^* (unilateral optic nerve aplasia) are missense mutations affecting nuclear localization of mutant proteins (Ragge et al., 2005; Gorbenko Del Blanco et al., 2012). Functional assays indicate that *OTX2^P134R^* mutation is dominant and produces the mutant protein with reduced transactivation activity. Mutation proteins produced by *OTX2^P133T^* and *OTX2^P134A^* have normal transactivation activity (Chatelain et al., 2006). It is unclear if mutant proteins still function in the nucleus and how the dominant-negative effect of *OTX2^P134R^* mutation contributes to the disease phenotypes. A large majority of nonsense or frameshift mutations within the coding region for transactivation domain cause reduced or loss-of-function transactivation, including *OTX2^S135LfsX2^* (bilateral optic nerve aplasia), *OTX2^S136LfsX43^* (bilateral optic nerve aplasia), *OTX2^S138X^* [Leber congenital amaurosis (LCA) or retinal dystrophy], *OTX2^S167X^* (bilateral microphthalmia), *OTX2^C170X^* (retinal dystrophy), *OTX2^Y179X^* (bilateral microphthalmia) and *OTX2^G188X^* (bilateral microphthalmia) (Ragge et al., 2005; Henderson et al., 2009; Tajima et al., 2009; Ashkenazi-Hoffnung et al., 2010; Gregory et al., 2021). These mutations are thought of having intact DNA-binding specificity and showing no dominant-negative effect. *OTX2^Y179X^* causes nearly lost transactivation, while *OTX2^G188X^* (only 8 aa apart) has 50% reduction. Both causes microphthalmia with many similar manifestations, suggesting high-level (>50%) *OTX2* expression is essential to eye development. Detailed functional assays with graded *OTX2* expression may help to address this hypothesis. Despite *OTX2^S138X^*, *OTX2^C170X^* and *OTX2^Y179X^* producing no transactivation activity, the disease phenotypes are associated with retinal defects instead of microphthalmia or anophthalmia. It is worth noting that various cases of incomplete penetrance have been reported in patient families (Ragge et al., 2005; Wyatt et al., 2008; Ashkenazi-Hoffnung et al., 2010; Schilter et al., 2011), including patients with *OTX2^Y179X^*. Regardless of possible phenotypic variations by incomplete penetrance, further studies need to determine how these mutations specifically affect the retina.

Lastly, sporadic, *de novo* and familiar *OTX2* mutations with complete penetrance account for 37, 42, 16% of reported cases, respectively (Fang et al., 2016). Patients with *OTX2* mutations usually develop pituitary hormone deficiency. The frequency of co-existence of pituitary hormone deficiency with ocular defects is however unclear. There are few cases with pituitary dysfunction without an ocular phenotype: *OTX2^R127W^* and *OTX2^N233S^* (Diaczok et al., 2008; Matsumoto et al., 2020). Furthermore, non-coding regions such as *DHS-4* are required to initiate *Otx2* expression (Emerson and Cepko, 2011; Muranishi et al., 2011; Wilken et al., 2015; Chan et al., 2020), mutation within these regions has not yet been reported in humans. Due to the complex pathogenetic mechanisms, treatment to *OTX2* mutations is currently unavailable.

#### CRX

**Figure 2:**
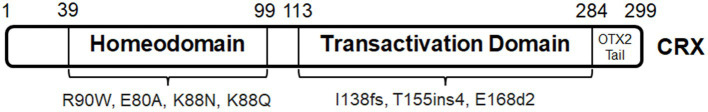
CRX protein domains and associated mutations.

*CRX* is another homeobox gene that is located on human chromosome 19 and expressed in vertebrate photoreceptors and some bipolar cells as well as in pineal gland (Chen et al., 1997; Furukawa et al., 1997, 1999; Rovsing et al., 2011). CRX function in photoreceptor development is briefly introduced in Table 1. The CRX protein consists of three major domains: the homeodomain at residues 39–99 facilitates the DNA binding; the transactivation domain at residues 113–284, including a WSP motif at residues 158–170, contains binding sites for other transcription coregulators; conserved OTX tail is found at resides 284–295 (Figure 2; Freund et al., 1997; Tran et al., 2014).

Pathogenic *CRX* mutations are associated with macular dystrophy (Hull et al., 2014), cone-rod dystrophy (CRD) (Freund et al., 1997), retinitis pigmentosa (RP) (Sohocki et al., 2001), and LCA (Freund et al., 1998; Rivolta et al., 2001). *CRX* mutations are known to occur *de novo* or to be inherited mostly in an autosomal dominant pattern, consisting of nonsense, missense, and frameshift mutations (Stenson et al., 2014). *CRX* mutations could cause dominant disorders by two possible mechanisms, namely, the *CRX* haploinsufficiency, and/or dominant negative or gain-of-function effects of the mutant proteins. Unlike *OTX2* mutations, *CRX* haploinsufficiency may not cause severe phenotypes. The study on *Crx*^+/−^ mice do not develop any detectable functional defects up to 6 months (Tran and Chen, 2014). Human patients with *CRX* heterozygosity do not develop LCA either (patients with *CRX* nullizygosity develop LCA) (Ibrahim et al., 2018). Therefore, the dominant-negative effects are ascribed to the functions of mutant proteins. However, it remains unknown if the mutant *CRX* allele could partially abrogate the production of a functional CRX from the normal allele. Further studies are needed to address this question in detail. Alternatively, dominant negative activities of mutant proteins have been demonstrated in animal models (Tran and Chen, 2014; Ruzycki et al., 2017). The reported dominant-negative mutations that arise in the homeodomain are mostly missense mutations, and those identified in the transactivation domain are largely frameshifts (Rivolta et al., 2001; Tran and Chen, 2014). Various knockin mouse models harboring mutations identified in human patients have been generated for the pathogenic analysis.

*CRX^R90W^* presents a hypomorphic missense mutation located in the homeodomain (Swaroop et al., 1999; Tran et al., 2014), and is associated with a dominant late-onset mild CRD and recessive LCA. The mutant protein has abolished DNA binding activity, and thus cannot transactivate target genes (Swaroop et al., 1999; Tran et al., 2014). *CRX^E80A^* and *CRX^K88N^* mutations represent distinct antimorphic missense mutations located in the homeodomain (Chen et al., 2002; Nichols II et al., 2010; Terrell et al., 2012), which manifest early-onset dominant CRD and dominant LCA in human patients, respectively (Freund et al., 1997; Nichols II et al., 2010). These mutant proteins are predicted to bind discrete DNA sequences and show different transactivation activities from the wildtype control. Future animal model studies will provide insights into the pathogenesis of these dominant mutations.

*CRX^E168d2^* presents an antimorphic frameshift mutation located in the transactivation domain (Tran et al., 2014), and is associated with dominant LCA in human patients (Freund et al., 1998; Jacobson et al., 1998). This mutation results in the early truncation of the transactivation domain, producing a protein that retains the ability of DNA binding but fails to transactivate target genes (Tran et al., 2014). In addition, *CRX^E168d2^* allele overproduces the mutant protein at about four times more than the wildtype protein in heterozygous mice, which exacerbates the dominant-negative effect on the binding competition (Tran et al., 2014). Cone photoreceptor degeneration occurs prior to rod photoreceptor degeneration in the heterozygous mice, whereas rod photoreceptor appears functional with shorter outer segments at 1 month-old but undergoes progressive cell death till complete loss at 6 month-old (Tran et al., 2014). Interestingly, the ratio of mutant to wildtype CRX proteins directly correlates with the disease phenotype severity (Tran et al., 2014). In addition, truncation at the last exon by frameshift results in premature terminations of transcription (Rivolta et al., 2001; Stenson et al., 2014), producing shortened but stable mutant mRNA that may avoid nonsense-mediated decay (Lejeune and Maquat, 2005). *Crx^Rip^* presents a unique mouse model with the c.763del1 mutation located in the last exon, causing a skipping of the OTX tail and a non-homologous extension of 133 residues (Roger et al., 2014). The mutant protein does not bind or transactivate target genes (Roger et al., 2014). *Crx^Rip/+^* mice show LCA-like phenotypes (Roger et al., 2014). Photoreceptors in *Crx^Rip/+^* mice do not form outer segments, due to impaired photoreceptor gene expression and incomplete differentiation at early development (Roger et al., 2014). The dominant-negative effect of *Crx^Rip^* mutation does not signify a competition between the mutant and WT proteins, but likely arises from the disruption of the photoreceptor gene expression network.

*AAV-based CRX* gene augmentation can partially rescue the photoreceptor phenotypes and restore expression of phototransduction-related genes in *CRX^K88N^* or *CRX^I138fs48^* human retinal organoids (Kruczek et al., 2021). On the other hand, knockout of *CRX* mutant alleles by CRISPR/Cas9-based gene editing can achieve moderate rescue of photoreceptor phenotypes in *CRX^K88Q/+^* or *CRX^T155ins4/+^* retinal organoids (Chirco et al., 2021). Thus, both gene augmentation and gene-editing-based therapies have translational potential to treat early-onset *CRX*-associated retinopathies.

#### NEUROD1

NEUROD1 is a basic helix–loop–helix (bHLH) transcription factor regulating the development of the cerebellum, hippocampal dentate gyrus, olfactory system, inner ear and auditory system, retina, and endocrine pancreas; it forms heterodimers with other bHLH transcription factors and binds to E box-containing promoter sequences to regulate gene expression of target genes (Naya et al., 1997; Poulin et al., 1997; Miyata et al., 1999; Liu et al., 2000; Breslin et al., 2003; Bernardo et al., 2008; Pan et al., 2009; Boutin et al., 2010; Evsen et al., 2013; Mastracci et al., 2013). *NEUROD1* is located on human chromosome 2 and well-known of regulating β-cell development, insulin synthesis and secretion, as well as glucose homeostasis (Huang et al., 2002; Petersen et al., 2002; Andrali et al., 2007; Romer et al., 2019). *NEUROD1* inactivation during the differentiation of human embryonic stem cells causes neonatal diabetes mellitus and defective β-cell function (Romer et al., 2019). Early-onset diabetes due to homozygous or heterozygous *NEUROD1* mutations have also been reported in human patients (Kristinsson et al., 2001; Liu et al., 2007; Gonsorčíková et al., 2008; Rubio-Cabezas et al., 2010; Chapla et al., 2015; Bouillet et al., 2020; Brodosi et al., 2021), thus *NEUROD1* is associated with maturity-onset diabetes of the young (MODY), i.e., MODY6 (Horikawa and Enya, 2019). Heterozygous *NEUROD1* mutations are also linked to autosomal dominant type 2 diabetes (Malecki et al., 1999, 2003).

**Figure 3:**
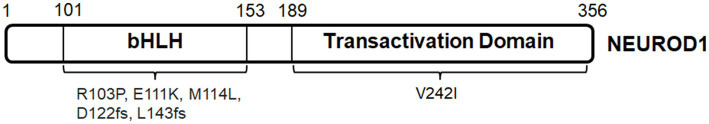
NEUROD1 protein domains and associated mutations.

NEUROD1 function in photoreceptor development is briefly introduced in Table 1. Ophthalmological records of patients with *NEUROD1* mutations are limited (Figure 3). Patients with homozygous frameshift mutations (*NEUROD1^D122GfsX12^* and *NEUROD1^L143AfsX55^*) develop permanent neonatal diabetes and neurological abnormalities including retinal disorders (Rubio-Cabezas et al., 2010; Orosz et al., 2015). The truncated mutant proteins are considered of lacking the transactivation domain for transcription regulatory functions. Patients with *NEUROD1^L143AfsX55^* develop nyctalopia, blurry vision, and visual field constriction from early childhood, and show absent rod- and cone-driven ERG responses (Orosz et al., 2015). These manifestations are similar to those caused by RP and rod–cone dystrophy (RCD). Interestingly, homozygous missense mutation *NEUROD1^V242I^* is associated with non-syndromic autosomal recessive RP (Wang et al., 2014). This mutation happens within the coding region for the transactivation domain. Since patients do not develop early-onset defects, this mutation might only affect the functional maintenance of photoreceptors in adulthood. These findings suggest differences in the functional roles of human NEUROD1 and mouse counterpart. Human NEUROD1 transactivation domain, at least a subdomain, is essential for photoreceptor development, while mouse NEUROD1 is required for functional maintenance. In addition, a bioinformatic analysis shows that *NEUROD1* is differentially expressed at optic nerve head of patients with primary open-angle glaucoma; histological evidence and patient cases have not been documented (Wang et al., 2017).

The conserved bHLH domain is located at aa101–153. Mutations within the region coding bHLH, including *NEUROD1^R103P^*, *NEUROD1^E111K^* and *NEUROD1^M114L^*, probably abolish the binding of the mutant proteins to the promoters of target genes (Kristinsson et al., 2001; Szopa et al., 2016; Brodosi et al., 2021). These mutations are associated to MODY. However, ophthalmological records of patients with these mutations are unavailable. Furthermore, considering NEUROD1’s important functions in glucose homeostasis, its role in the pathogenesis of diabetic retinopathy has not been reported.

*AAV*-based *NeuroD1*-mediated gene therapies can reprogram brain astrocytes into neurons that are able to re-establish synapses and integrate with the survived neurons after ischemic injury in mice (Chen et al., 2020; Wu et al., 2020; Tang et al., 2021). In particular, the reprogrammed neurons form specific projections and functional connectivity in the mouse primary visual cortex, promoting the recovery of visual responses and orientation discrimination (Tang et al., 2021). However, *NEUROD1*-mediated gene therapy has not been proposed in the retina.

#### NRL and *NR2E3*

NRL is a basic-motif leucine zipper transcription factor that is encoded by the gene on human chromosome 14 and expressed in developing lens, developing and mature rod photoreceptors and pineal gland (Swaroop et al., 1992; Liu et al., 1996; Farjo et al., 1997; Swain et al., 2001; Kanda et al., 2007). NRL function in photoreceptor development is briefly introduced in Table 1.

**Figure 4:**
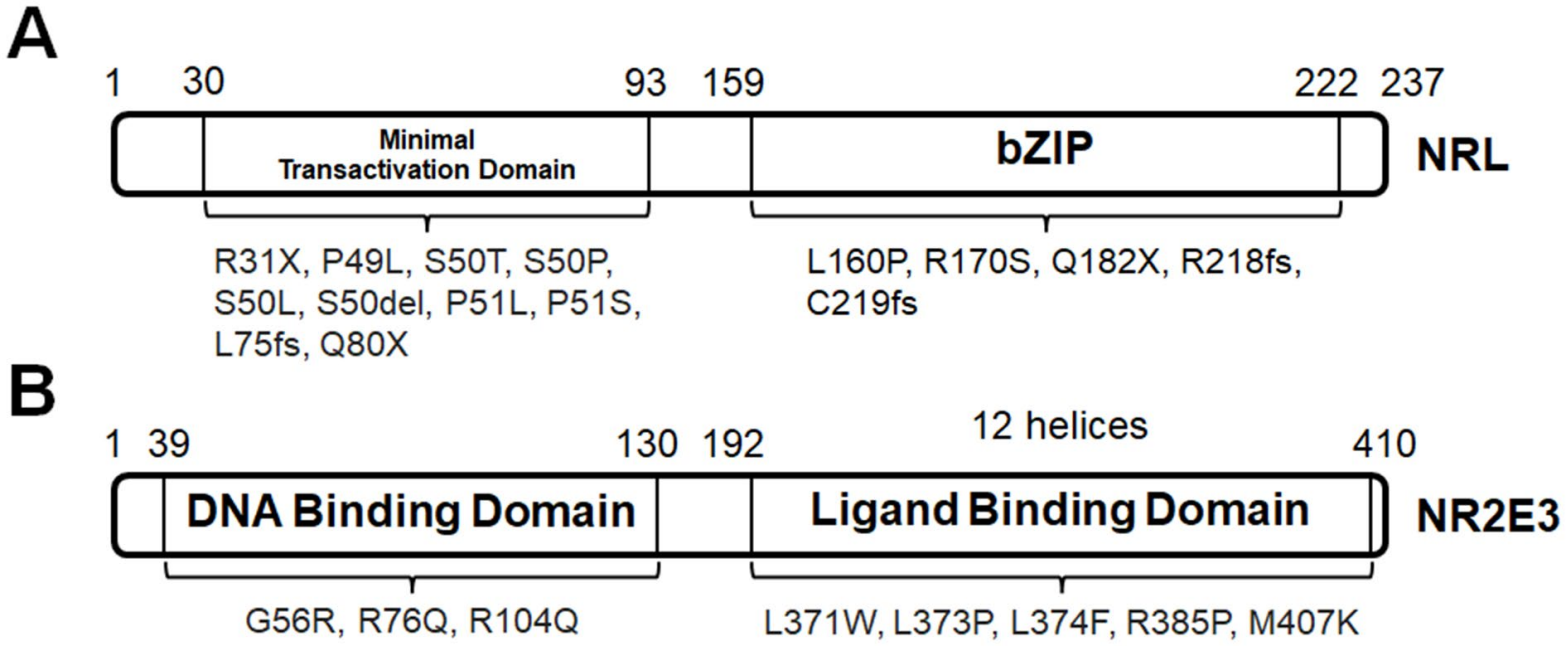
**(A)** NRL protein domains and associated mutations. **(B)** NR2E3 protein domains and associated mutations.

In general, night blindness from early childhood is a common symptom for patients with pathogenic *NRL* mutations, followed by variable onsets of reduced visual acuity. Mutations can be classified by the protein domains, namely, bZIP domain and minimal transactivation domain (MTD) (Figure 4A). NRL bZIP domain is located at aa159-222. Mutations within the coding region for bZIP domain, such as heterozygous missense mutation *NRL^L160P^* (compound with *NRL^A76GfsX18^*), homozygous missense mutation *NRL^R170S^*, homozygous nonsense mutation *NRL^Q182X^*, and homozygous frameshift mutations *NRL^R218fs^* and *NRL^C219fs^* affect DNA binding and transcription activation of target genes (Nishiguchi et al., 2004; Kanda et al., 2007; Collin et al., 2011; Neveling et al., 2012; Littink et al., 2018; El-Asrag et al., 2022). These mutations cause autosomal recessive RP, some of which are specified as clumped pigment retinal degeneration that is manifested by clusters of pigmented deposits at the peripheral retina, chorioretinal atrophy and attenuated arterioles (Newman et al., 2016; Littink et al., 2018). Autosomal dominant mutation within bZIP-coding region has not been reported. NRL MTD is located at aa30-93. Homozygous mutations within MTD-coding region, including *NRL^R31X^*, *NRL^L75fs^,* and *NRL^Q80X^* (Kanda et al., 2007; Newman et al., 2016; El-Asrag et al., 2022), are considered of lacking bZIP domain. In particular, *NRL^R31X^* results in an early truncation of the mutant protein at MTD and causes enhanced S-cone syndrome (ESCS) with no detectable rod-driven ERG response, which matches retinal phenotypes in *Nrl−/−* mice (Newman et al., 2016). However, the screening analysis in cohorts of ESCS patients indicates that *NRL* mutation is a rare cause (Acar et al., 2003; Nishiguchi et al., 2004; Wright et al., 2004a; Collin et al., 2011; Neveling et al., 2012). The rest of pathogenic mutations within MTD-coding region belong to the autosomal dominant class, including *NRL^P49L^* and mutations at hot spots S50 (*NRL^S50T^*, *NRL^S50P^*, *NRL^S50L^,* and *NRL^S50del^*) and P51 (*NRL^P51L^* and *NRL^P51S^*) (Martinez-Gimeno et al., 2001; DeAngelis et al., 2002; Kanda et al., 2007; Gao et al., 2016; Qin et al., 2017; Mizobuchi et al., 2022). Interestingly, functional assays indicate that these missense mutations produce mutant proteins that have reduced level of phosphorylation but are able to enhance transcription activation at *Rho* promoter (Bessant et al., 1999; DeAngelis et al., 2002; Kanda et al., 2007). In humans, these mutations cause autosomal dominant RP with the signature phenotype of bone spicule–shaped pigment deposits. A significant number of patients with autosomal dominant mutations in *NRL* develop RP at adult ages, although only less than 30 cases have been reported so far.

*Nrl* knockout after the completion of photoreceptor fate determination may favor photoreceptor survival in mouse models of *Rho−/−*, *rd10* and *RHO^P347S^* (Montana et al., 2013; Yu and Wu, 2018). It is worth noting that *Rho* and *Pde6β* are direct target genes of NRL. Only a small population of differentially expressed genes between rod and cone photoreceptors significantly change their expression by *Nrl* knockout, including *Nr2e3* (Yu and Wu, 2018). More importantly, *Nrl* knockout in young adult mice does not produce retinal rosettes, Müller glia dysfunction and vascular defects which can be found in *Nrl−/−* retina (Roger et al., 2012; Yu and Wu, 2018). Therefore, *NRL* knockout can potentially serve as a neuroprotective method to preserve rod photoreceptors from ongoing degeneration without any significant disruption in transcription, structural and functional homeostasis.

A notable downstream target gene of NRL is *NR2E3* that is located on human chromosome 15. NR2E3 function in photoreceptor development is briefly introduced in Table 1. ESCS is exclusively associated with autosomal recessive mutations in *NR2E3*, for example, *NR2E3^R76Q^*, *NR2E3^R104Q^*, *NR2E3^L371W^*, *NR2E3^L373P^*, *NR2E3^L374F^*, *NR2E3^R385P^,* and *NR2E3^M407K^*, explaining over 90% of reported cases (Figure 4B; Wright et al., 2004a; Audo et al., 2008; Tsang and Sharma, 2018; de Carvalho et al., 2021). A large number of ESCS-associated mutations are located within the region coding the ligand-binding domain, especially the α-helix (Pachydaki et al., 2009; Tan et al., 2013). ESCS is often diagnosed through the typical features on ERG responses: loss of rod-driven response and increased S-cone-driven response (Vincent et al., 2013; Tsang and Sharma, 2018; de Carvalho et al., 2021). ESCS patients always suffer from nyctalopia at the first decade. They also develop clumped pigment deposits at RPE, dot-like lesions at ONL, and variable loss of visual acuity (Jacobson et al., 1990, 1991; Audo et al., 2008; Garafalo et al., 2018; Tsang and Sharma, 2018). Notably, hyper-sensitivity of S-cone photoreceptors at early onset concentrates at the central field and extends into the peripheral field.

In-depth analysis of ERG and phenotypic findings with ESCS patients at various disease stages suggests a parallel pattern between disease manifestations and observations in *rd7* mice (Wright et al., 2004b; Iannaccone et al., 2021). However, differences between human patients and *rd7* mice are noteworthy. Firstly, rod-driven ERG response is still detectable in young *rd7* mice (Akhmedov et al., 2000; Ueno et al., 2005). ESCS patients show loss of rod-driven ERG response at early childhood. Secondly, ESCS patients only have dysplastic photoreceptors or pseudo-rosettes at ONL, as compared to the more deleterious structure of whorls and rosettes at ONL in *rd7* mice (Wang et al., 2009). Lastly, such parallel pattern between ESCS patients and *rd7* mice is limited to functional and histological measurements; comparative gene expression profiles have not been documented.

Pathogenic *NR2E3* mutations are also associated with autosomal recessive (Gerber et al., 2000; Tan et al., 2013; Al-khuzaei et al., 2020) and autosomal dominant RP (*NR2E3^G65R^*) (Coppieters et al., 2007), although only a few cases have been reported. In terms of treatment strategies to *NR2E3*-associated retinopathies, fate-switch to developmentally altered photoreceptors might be unrealistic. Thus, practical approaches aim to slow down the progression of retinal degeneration. An *in vitro* study proposes a treatment strategy of knocking down a *NR2E3* pathogenic variant by antisense oligonucleotides (Naessens et al., 2019). In addition, *in vivo* treatment by photoregulin-3 (PR3), a NR2E3 inhibitor, can slow down the photoreceptor degeneration in *Rho^P23H^* mice (Nakamura et al., 2017). Therefore, these findings suggest *NR2E3* antagonism helps to reduce susceptibility of rod photoreceptors to genetic insults possibly by conferring cone photoreceptor properties. Interestingly, *NR2E3* as a genetic modifier directly can serve as a therapeutic target to treat inherited retinal diseases including *NR2E3*-associated retinopathies. *Nr2e3* overexpression yields promising rescue results in mouse models of *rd1*, *rd7*, *rd16*, *Rho^−/−^,* and *Rho^P23H^* (Li et al., 2021): *AAV8-Nr2e3* helps to preserve photoreceptor density, promote cell survival at ONL, and enhance ERG responses. The therapeutic mechanisms of *NR2E3* antagonism and overexpression are subjected to further investigation.

#### THRB and *RXRG*

Cones with different wavelength sensitivities develop from RPCs and subsequently differentiate for distinct color perceptions, which is reliant on specific transcription factors. THRB and RXRG are two notable transcription factors for this process.

Thyroid hormone receptors are a family of ligand-dependent nuclear receptors, characterized by the conserved protein structure of an N-terminus, a DNA binding domain that binds to the *thyroid hormone response elements* (*TREs*), and a ligand binding domain for triiodothyronine (T3) across many vertebrate species including zebrafish, chicken, mouse, and human (Sjöberg and Vennström, 1995; Deeb, 2006; Darras et al., 2011; Ng et al., 2011). T3 is important for many body functions including metabolism, heart rate, and tissue development (Li et al., 2014; Mullur et al., 2014; Bassett and Williams, 2016; Chattergoon, 2019; Vale et al., 2019; Bernal et al., 2022). THRA and THRB are two members of this family (Forrest et al., 2002). *THRA* is located on human chromosome 17, while *THRB* is located on human chromosome 14. THRB function in photoreceptor development is briefly introduced in Table 1. In particular, *THRB isoform 2*, *THRB2* (also known as *TRβ2*) is expressed in cone photoreceptors (Sjöberg et al., 1992; Applebury et al., 2007; Ng et al., 2009; Suzuki et al., 2013; Marelli et al., 2016). Zebrafish is a useful model for understanding the roles of thyroid hormone signaling and *trβ2* in cone photoreceptor development. Firstly, Trβ2 binds to activate its own *trβ2* promoter, suggesting that *trβ2* expression is self-regulating (Suzuki et al., 2013). Secondly, Trβ2 determines the fate and proper L-cone differentiation and regulates the expression of opsins (*opn1lw1* and *opn1lw2*) (Suzuki et al., 2013; Volkov et al., 2020). Samples with ablated thyroid glands maintain a similar level of *opn1lw2* expression as the *WT* controls during development, suggesting that Trβ2 regulates L-cone differentiation independent of thyroid hormones (Mackin et al., 2019). Thirdly, Trβ2 may not be involved in the establishment of cone density ratio during development (Deveau et al., 2020). Lastly, Trβ2 regulates Cyp27c1 expression in zebrafish RPE for the production of vitamin A2-based retinoids, implying that Trβ2 signaling may interact with other signaling pathways to promote retinal development (Volkov et al., 2020). Fate switch of L-cone precursors to UV cones in *thrb*^−/−^ zebrafish retina generally agrees with the selective changes in *Thrb2^−/−^* mouse retina, i.e., decrease in *Opn1mw* expression and increase in *Opn1sw* expression, supporting a conserved developmental role (Ng et al., 2001; Volkov et al., 2020).

**Figure 5:**
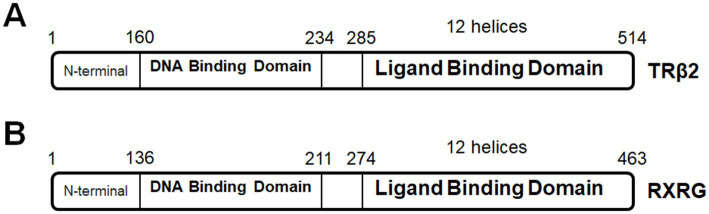
**(A)** TRβ2 protein domains. **(B)** RXRG protein domains.

In general, heterozygous *THRB* mutations are associated with a metabolic syndrome called resistance to thyroid hormone beta (RTHβ) (Onigata and Szinnai, 2014; Concolino et al., 2019; Pappa, 2021). More than 200 different *THRB* mutations have already been identified in RTHβ patients. A large number of these mutations happen at the coding region for ligand-binding domain and hinge region (Pappa and Refetoff, 2018), inhibiting TRβ2 binding as homodimers to TREs (Figure 5A). Detailed ophthalmological records of RTHβ patients are infrequent. Of particular interest, a study of clinical observations with 31 RTHβ patients concludes functional defects in RTHβ photoreceptors and deficits in color vision (Campi et al., 2017). Another case report shows that a child with a compound missense *THRβ^R338W/R429W^* mutation at the coding region for ligand-binding domain has severely reduced M- and L-cone-driven ERG responses and increased S-cone-driven ERG responses (Weiss et al., 2012). Unfortunately, treatment strategies targeting *THRB* mutations have not been developed for retinal defects.

Retinoid X receptor (RXR) belongs to the nuclear hormone superfamily that comprises three isoforms, namely, α, β, and γ. RXRs share a common protein structure: the N-terminal, a DNA-binding domain and a ligand-binding domain (Rowe, 1997; Dawson and Xia, 2012). RXRs form both homo- and hetero-dimers with a number of nuclear receptors, including thyroid hormone receptors, retinoic acid receptors, and peroxisome-proliferator-activated receptors (Mangelsdorf and Evans, 1995; Chawla et al., 2001), and bind to repeats of the consensus sequence AGGTCA with a 1 base pair spacer (Rowe, 1997).

*RXRG* is located on human chromosome 1 and expressed in the retina of several species, including human, mouse, chick, zebrafish, xenopus (Hoover et al., 1998; Janssen et al., 1999; Mori et al., 2001; Cossette and Drysdale, 2004; Roberts et al., 2005; Stevens et al., 2011). RXRG function in photoreceptor development is briefly introduced in Table 1. RXRG deficiency can cause metabolic disorders, including type 2 diabetes (Brown et al., 2000; Davies et al., 2001; Wang et al., 2002; Haugen et al., 2004). A mutation in the coding region for helix–helix interface could impair various cellular processes (Zhang et al., 2004; Figure 5B); unfortunately, retina-specific *RXRG* mutation has not been reported yet. Interestingly, RXRG can serve as a therapeutic target for retinopathies. RXR agonist PA024 can selectively upregulate *Rxrg* expression and decrease photoreceptor cell death in mixed neuro-glial cultures from *rd1* retinas (Volonté et al., 2021).

### Other transcription factors

Inherited retinal diseased can be caused by other transcription factors, such as *AHR* (Zhou et al., 2018), *ATF6* (Xu et al., 2015; Chiang et al., 2019), *RORB* (Sadleir et al., 2020; Morea et al., 2021). It is worth mentioning that disease-associated mutations do not always produce loss-of-function variants, two examples as follows.

#### PRDM13

North Carolina macular dystrophy (NCMD) is an inheritable abnormality affecting the macula, which usually occurs at birth but progresses little with aging. NCMD is inherited as an autosomal dominant manner and completely penetrant with phenotypic variability. Intragenic mutations in *PRDM13* gene have not been reported for NCMD. However, a number of NCMD patients carry missense mutations in the MCDR1 locus upstream of *PRDM13* gene in human chromosome 6 (Small et al., 2016, 2019a,b; Namburi et al., 2020). These mutations may alter the spatio-temporal pattern of *PRMD13* expression. In the eye, *PRDM13* is expressed in the fetal (Small et al., 2016) and adult retina (Green et al., 2021), predominantly in amacrine cells. In particular, *PRDM13* regulates the development and subtype specification of amacrine cells in xenopus and mouse retinas (Watanabe et al., 2015; Bessodes et al., 2017). Interestingly, the sequencing analysis on a family of NCMD patients shows a tandem duplication of *PRDM13* gene and a partial copy of *CCNC* gene in MCDR1 locus, suggesting *PRDM13* overexpression responsible for NCMD pathogenesis (Bowne et al., 2016). A similar case of *PRDM13* duplication also reports NCMD phenotype (Small et al., 2021). Indeed, *CG13296* (*PRDM13* orthologue) overexpression severely affects the development of eye-antennal imaginal disks in *Drosophila melanogaster* (Manes et al., 2017). In addition, a single nucleotide variant located 7.8 kb upstream of *PRDM13* gene (within the MCDR1 locus) is associated with autosomal dominant progressive bifocal chorioretinal atrophy that is presumably related to NCMD (Silva et al., 2019). The regulatory function of the MCDR1 locus remains to be determined.

#### RAX2

RAX2 interacts and synergistically functions with CRX (Wang et al., 2004), and is required for photoreceptor differentiation in vertebrate retina (Chen and Cepko, 2002; Nelson et al., 2009; Wu et al., 2009; Irie et al., 2015). Pathogenic variants in *RAX2* (human chromosome 19) cause autosomal dominant retinal dystrophies, including CRD, RP and age-related macular degeneration (Wang et al., 2004; Yang et al., 2015; Van de Sompele et al., 2019). Surprisingly, increased transactivation activity has been observed in *in vitro* functional analysis on *RAX2* mutations (Wang et al., 2004), such as *RAX2^R87Q^* and *RAX2^P140_G141dup^*. *RAX2^R87Q^* occurs in the coding region for homeodomain (aa 25–89), while *RAX2^P140_G141dup^* is found in the coding region for the transactivation domain. Other reported mutations including *RAX2^S49P^* (homozygous), *RAX2^P52R^* (heterozygous), RAX2^A113Gfs*178^ (homozygous), RAX2^A156Rfs*131^ (heterozygous), *RAX2^G137R^* (heterozygous) show reduced transactivation activity. Further disease modeling analysis will inform insights into the roles of RAX2 in transcriptional coactivation with other transcription factors, as well as functions of RAX2 in retinal development and pathogenesis.

## Conclusion

All in all, photoreceptor development is regulated by a specific network of transcription factors. Genetic variations in these genes result in autosomal recessive or dominant mutations. This review provides a mechanistic enlightenment of the genotype–phenotype relationship between above-mentioned mutations and ocular disease manifestations. In general, *in vitro* or *in vivo* functional analysis of the mutant proteins helps to determine their conformational changes, regulatory capacity, and interference with the action of wildtype proteins, which can be further correlated to the functional roles of specific protein domains. Thus, missense, nonsense and frameshift mutations that happen to the same coding region may produce mutant proteins with different regulatory functions. When the animal model is unavailable for a specific mutation, such as cases of *OTX2* mutations, genotype–phenotype relationship would solely reply on *in vitro* molecular analysis. **T**he study of animal models is conducive to understanding the pathogenic mechanisms of blindness-causing mutations, as well as testing therapeutic approaches. The use of animal models also helps to dissect the disease progression for cell-type specificity, expanding the scope of genotype–phenotype relationship; such examples can be found in *CRX*-associated retinopathies. Hence, understanding genotype–phenotype relationship benefits two horizons: (1) predictions on the disease onset/progression of an unknown mutation; (2) management of treatment windows. Gene therapy holds a promise in treating early-onset inherited retinal diseases, although significant challenges and unanswered knowledge gaps remain. A long-overlooked issue is how effective a strategy of gene therapy such as gene augmentation can treat an unknown mutation. In order to tackle this issue, genotype–phenotype relationship needs to fulfill excellent predictive power. In-depth analysis of domain-based transcription factor interactome as well as mutant/wildtype protein binding motifs can collectively help to achieve this goal.

## Author contributions

CS conceived the contents, drafted the manuscript, and prepared the figures. SC edited the manuscript and figures. All authors contributed to the article and approved the submitted version.

## Funding

NIH grants R01 EY012543 and R01 EY032136 (to SC), and Research to Prevent Blindness (to DOVS).

## Conflict of interest

The authors declare that the research was conducted in the absence of any commercial or financial relationships that could be construed as a potential conflict of interest.

## Publisher’s note

All claims expressed in this article are solely those of the authors and do not necessarily represent those of their affiliated organizations, or those of the publisher, the editors and the reviewers. Any product that may be evaluated in this article, or claim that may be made by its manufacturer, is not guaranteed or endorsed by the publisher.
